# Association of age-related changes in circulating intermediary lipid metabolites, inflammatory and oxidative stress markers, and arterial stiffness in middle-aged men

**DOI:** 10.1007/s11357-012-9454-2

**Published:** 2012-07-18

**Authors:** Ji Young Kim, Oh Yoen Kim, Jean Kyung Paik, Dae Young Kwon, Hyun-Jin Kim, Jong Ho Lee

**Affiliations:** 1 Yonsei University Research Institute of Science for Aging, Yonsei University, Seoul, Korea; 2 Department of Culinary Nutrition, Woosong University, Daejeon, Korea; 3 Department of Food Science and Nutrition, College of Human Ecology, Dong-A University, Busan, Korea; 4 Research Laboratory of Clinical Nutrigenetics/Nutrigenomics, Department of Food and Nutrition, College of Human Ecology, Yonsei University, 134 Shinchon-Dong, Sudaemun-Gu, Seoul, 120-749 Korea; 5 Emerging Innovative Technology Research Division, Korean Food Research Institutes, Daejon, Korea

**Keywords:** Age-related changes, Intermediate metabolites, Inflammation, Oxidative stress, Arterial stiffness

## Abstract

The relationships between age-related changes in circulating endogenous metabolites, inflammatory and oxidative stress markers, and arterial stiffness in 57 middle-aged (34–55 years), nonobese men were studied over the course of 3 years. Arterial stiffness was measured using brachial-ankle pulse wave velocities (ba-PWV). Plasma metabolomic profiling was performed using ultra-performance liquid chromatography and quadrupole time-of-flight mass spectrometry. After 3 years, decreased HDL cholesterol and increased malondialdehyde (MDA) and ox-LDL levels were observed. Among 15 identified lipids, lysoPCs (C16:0, C18:0, C18:2, C20:4, and C20:5) and linoleyl carnitine were the major plasma metabolites that contributed to the age-related differences. LysoPC16:0 (variable importance in the projection value, 6.2029) was found as the most important plasma metabolite for evaluating these changes. Changes in lysoPC16:0 levels positively correlated with the changes in 8-epi-PGF_2α_ (*r* = 0.608), MDA (*r* = 0.413), high-sensitivity C-reactive protein (*r* = 0.509), IL-6 (*r* = 0.497), and ba-PWV (*r* = 0.283) levels. ba-PWV levels positively correlated with the changes in waist-to-hip ratios (WHR), inflammatory and oxidative stress markers. In a subgroup analysis of subjects with decreased ba-PWVs vs. increased ba-PWVs, changes in WHR and levels of lysoPC16:0, ba-PWV, IL-6, 8-epi-PGF_2α_, MDA, and P-selectin were significantly different. Our results suggest that age-related increases in lysoPC16:0 may contribute to lipid peroxidation, thereby activating proinflammatory phenotypes and arterial stiffness.

## Introduction

Aging and increased levels of circulating proinflammatory markers and oxidized LDLs (ox-LDLs) are associated with arterial stiffness (Kampus et al. 2007; Byfield et al. 2006; Brinkley et al. 2009; Scuteri et al. 2011). In particular, arterial stiffness increases with age, even in healthy individuals without clinical cardiovascular disease (CVD) (Zieman et al. 2005). Although the exact mechanism of age-related arterial stiffness is not fully understood, changes in numerous endogenous metabolites in a complex physiological aging process (Nevedomskaya et al. 2010; Yan et al. 2009) could partly result in increases of proinflammation, oxidative stress, and arterial stiffness. Particularly, lysophosphatidylcholines (lysoPCs), proinflammatory lipid mediators, are generated from phospholipase A_2_ (PLA_2_)-catalyzed hydrolysis of phosphatidylcholine. In addition, lysoPCs are reported to constitute only 1–5 % of the total PC content of non-ox-LDL; however, about 40–50 % of PC contained within LDL molecules is converted to lysoPC during LDL oxidation (Matsumoto et al. 2007). LysoPCs are also formed by the action of lecithin cholesterol acyltransferase in plasma (Kougias et al. 2006; Matsumoto et al. 2007). Recently, the inflammation induced by saturated or mono-unsaturated acyl-lysoPC in vitro has been well established. Therefore, understanding of age-related changes in these metabolites and their relationships with inflammatory and oxidative stress markers will allow better understanding of the pathological or physiological processes underlying arterial stiffness, a condition closely associated with aging.

Accordingly, to study the relationship of age-related changes in the levels of endogenous metabolites and inflammatory and oxidative stress markers with arterial stiffness, we observed 57 nonobese men between the ages of 34 and 55 years, without a disease history for 3 years. To compare age-related changes between the baseline and 3-year follow-up data, we used a metabolomics approach based on the combination of ultra-performance liquid chromatography and quadrupole time-of-flight mass spectrometry (UPLC-Q-TOF MS) coupled with multivariate data analyses. Additionally, to analyze the oxidative and inflammatory status of the study subjects at baseline and after 3 years, we measured the levels of inflammatory and oxidative markers using specific immunoassays and measured arterial stiffness using brachial-ankle pulse wave velocity (ba-PWV).

## Experimental

### Subjects

The study protocol was approved by the Institutional Review Board of the National Health Insurance Corporation (NHIC)-sponsored Ilsan Hospital, Korea, and was conducted in accordance with the Helsinki Declaration. Fifty-seven healthy, nonobese male subjects (20 ≤ body mass index (BMI) < 30 kg/m^2^) between the ages of 34–55 years visiting a health promotion center at the NHIC-sponsored Ilsan Hospital in Korea between August 2007 and October 2007 were enrolled in this study. The subjects led a sedentary lifestyle and had not participated in weight reduction programs within the previous 3 years. The subjects also completed a personal health and medical history questionnaire that served as a screening tool for enrollment. Exclusion criteria included the presence of type 2 diabetes, CVD, or psychiatric problems, or the use of anti-hypertensive, lipid-lowering, anti-platelet, or anti-diabetic medications. The duration of the study was 3 years. At baseline, the usual dietary intake of the study subjects was assessed using a semiquantitative food frequency questionnaire and a 24-h recall method. The subjects were encouraged to maintain their body weight within ±3 kg and were given general oral and written information about healthy food choices and exercise at baseline and at the subsequent visit (week 4). The subjects were instructed by trained dietitians and were also asked to keep 3-day food records (2 weekdays and 1 weekend) at each visit. Nutrient intake was determined and calculated based on the 3-day food records using the Computer-Aided Nutritional Analysis Program (CAN-pro 2.0; Korean Nutrition Society, Seoul, Korea). Total energy expenditure (TEE) (kcal/day) was calculated based on the activity patterns of the study subjects, such as basal metabolic rate, 24-h physical activity, and specific dynamic actions of food.

### Anthropometric parameters, blood pressure, and blood collection

Body weights and heights were measured in the morning while the study subjects were unclothed and without shoes. BMI (kg/m^2^) was calculated based on body weight and height. Percent body fat was analyzed using a TBF-105 body fat analyzer (Tanita Co., Tokyo, Japan). Waist circumference was measured at the umbilical level, with the subjects standing after normal expiration. Blood pressure (BP) was measured in the left arm of seated patients using an automatic blood pressure monitor (TM-2654, A&D, Tokyo, Japan) after a 20-min rest period. After a 12-h fast, venous blood specimens were collected in EDTA-treated or untreated tubes. Plasma or sera were separated and stored at −70 °C until further analysis.

### Serum lipid profiles and fasting glucose levels, insulin concentrations, and homeostasis model assessment for insulin resistance

Fasting total cholesterol and triglyceride levels were measured using commercially available kits and a Hitachi 7150 autoanalyzer (Hitachi Ltd., Tokyo, Japan). After precipitation of serum chylomicrons using dextran sulfate magnesium, HDL cholesterol concentrations in the supernatants were enzymatically measured. For subjects with serum triglyceride levels <400 mg/dL, LDL cholesterol levels were estimated directly using the Friedwald formula: LDL cholesterol = total cholesterol − (HDL cholesterol + [triglycerides/5]). For subjects with serum triglyceride levels ≥400 mg/dL, LDL cholesterol levels were measured indirectly. Fasting glucose levels were measured by the glucose oxidase method using a Beckman glucose analyzer (Beckman Instruments, Irvine, CA, USA). Insulin levels were measured by radioimmunoassay using a commercial kit (Immuno Nucleo Corporation, Stillwater, MN, USA). Insulin resistance (IR) was calculated based on the homeostasis model assessment (HOMA) using the following equation: HOMA-IR = (fasting insulin [μIU/mL] × fasting glucose [mmol/L])/22.5.

### Measurement of serum IL-6 levels, serum high-sensitivity C-reactive protein levels, white blood cell counts, and urinary 8-epi-PGF_2α_ levels

Serum interleukin (IL)-6 concentrations were measured using Bio-Plex™ Reagent Kits and a Bio-Plex™ system (Bio-Rad Laboratories, Hercules, CA, USA) according to the manufacturer’s instructions. The high-sensitivity C-reactive protein (hs-CRP) levels were measured on an Express Plus™ auto-analyzer (Chiron Diagnostics Co., Walpole, MA, USA) using commercially available high-sensitivity CRP-Latex (II) *X*2 kits (Seiken Laboratories Ltd., Tokyo, Japan). White blood cell (WBC) counts were determined using a hematology analyzer from HORIBA ABX Diagnostic (HORIBA ABX SAS, Parc Euromedicine, France). The compound 8-epi-PGF_2α_ was measured using an enzyme immunoassay (BIOXYTECH urinary 8-epi-PGF_2α_TM Assay kit, OXIS International Inc., Portland, OR, USA). Urinary creatinine levels were determined using the alkaline picrate (Jaffe) reaction.

### Plasma-oxidized LDL, adiponectin, malondialdehyde, sVCAM-1, sICAM-1, and P-selectin levels

Plasma-oxidized (ox) LDL levels were measured using an enzyme immunoassay (Mercodia, Uppsala, Sweden). Plasma adiponectin concentrations were measured using an enzyme immunoassay (Human Adiponectin ELISA kit, B-Bridge International Inc., CA, USA). The absorbencies of the resulting color reactions (ox-LDLs and adiponectin) were measured at a wavelength of 450 nm using a Wallac Victor^2^ multilabel counter (Perkin Elmer Life Sciences, Turku, Finland). The wavelength correction was set to 540 nm. Plasma malondialdehyde (MDA) concentrations were measured based on the production of thiobarbituric acid-reactive substances (TBARS Assay Kit, Zepto-Metrix Co., Buffalo, NY, USA). Plasma vascular cell adhesion molecule (VCAM)-1, inter-cellular adhesion molecule (ICAM)-1, and P-selectin levels were measured using Bio-Plex™ Reagent Kits with a Bio-Plex™ system (Bio-Rad) according to the manufacturer’s instructions.

### Brachial-ankle pulse wave velocity measurement

ba-PWVs were measured using an automatic waveform analyzer (model VP-1000; Nippon Colin Ltd., Komaki, Japan) according to a previously described method (Kim et al. 2010a, b). The average ba-PWV from both left and right sides was used for analysis (correlation between the right and left ba-PWVs: *r* ^2^ = 0.925, *P* < 0.001).

### Plasma metabolic profiling

Plasma samples were prepared and injected into a UPLC/Q-TOF MS (Waters, Milford, MA, USA) according to previously described methods (Kim et al. 2010a, b). The Q-TOF MS was operated in positive electrospray ionization (ESI) mode. The capillary and sampling cone voltages were set at 2.78 kV and 26 V, respectively. The desolvation flow was set to 700 L/h at 300 °C, and the source temperature was set to 110 °C. The TOF MS data were collected in the range of 50–1000 *m*/*z*, with a scan time of 0.2 s and interscan delay time of 0.02 s. All analyses were performed using lock spray to ensure accuracy and reproducibility; leucine-enkephalin (556.2771 Da in the positive ESI mode) was used as the lock mass at 200 pmol and a flow rate of 3 μL/min. The lock spray frequency was set at 10 s.

For quality control, a mixture of five standard compounds (caffeine, sulfadimethoxine, terfenadine, 4-acetoaminophenol, and reserpine) was injected after every seven samples. The MS/MS spectra of the metabolites were obtained by a collision energy ramp from 10 to 30 eV. Accurate masses and compositions of the precursor and fragment ions were calculated and sequenced using MassLynx 4.1 software (Waters) incorporated in the instrument. All MS data, including retention time, *m*/*z*, and ion intensity, were extracted using the MarkerLynx 4.1 software package (Waters) incorporated in the instrument, and the resulting MS data were assembled into a data matrix.

Peaks were collected using a peak width of 5 %, a height of 1 s, a noise elimination of 6, and an intensity threshold of 120. Data were aligned with a mass tolerance of 0.04 Da and a retention time window of 0.15 min. All spectra were aligned and normalized to the total peak intensity. Assignment of metabolites contributing to the observed variance was performed using the elemental composition analysis software using calculated mass, mass tolerance (mDa and ppm), double-bond equivalent, and the i-Fit algorithm (the likelihood that the isotopic pattern of the elemental composition matches a cluster of peaks in the spectrum) implemented in the MassLynx software by the ChemSpider database (www.chemspider.com) and by the Human Metabolome Database (www.hmdb.ca). Authentic standards were used to confirm the assignments and to perform quantitative analyses.

### Statistical analysis

Statistical analyses were performed using SPSS ver12.0 (Statistical Package for the Social Sciences, SPSS Inc., Chicago, IL, USA). The skewed variables were logarithmically transformed for statistical analysis. For descriptive purposes, mean values were presented using untransformed values. Results are expressed as means ± standard error (SE). A two-tailed *P* value of <0.05 was considered as statistically significant. Paired *t*-tests and Wilcoxon signed-rank tests were used to evaluate differences between baseline and 3-year follow-up levels. Differences in the clinical variables between the decreased ba-PWV and increased ba-PWV groups were tested by independent *t*-tests and Mann–Whitney *U*-tests. General linear model tests (mixed model tests) were applied to the comparison of the changes in variables over time by adjusting for confounding factors. Pearson’s and partial correlation coefficients were used to examine the relationships between variables over time.

Multivariate statistical analysis was performed using SIMCA-P^+^ software version 12.0 (Umetrics, Umeå, Sweden). Partial least-squares discriminant analysis (PLS-DA) was used as the classification method for modeling discrimination between the baseline and 3-year follow-up data by visualizing score plots or *S*-plots using the first and second PLS components. To validate the model, a sevenfold validation was applied to the PLS-DA model, and the reliabilities of the model were further rigorously validated by a permutation test (*n* = 200). Goodness of fit was quantified by R^2^Y, while the predictive ability was indicated by Q^2^Y. Generally, R^2^Y, which describes how well the data in the training set are mathematically reproduced, varies between 0 and 1, with 1 indicating a model with a perfect fit.

## Results

### Clinical characteristics, inflammatory markers, arterial stiffness, lipid peroxides, adhesion molecules, and nutrient intakes at baseline and at 3-year follow-up

After 3 years, the subjects showed decreased levels of HDL cholesterol (*P* < 0.001) and increased levels of MDA (*P* < 0.001) and ox-LDLs (*P* < 0.001) (Table 1). There were no significant differences in the levels of inflammatory markers, arterial stiffness, and adhesion molecules between the baseline and 3-year follow-up data. The estimated total calorie intake at baseline was 2,441 ± 27 kcal/day and at 3-years follow-up was 2,429 ± 23 kcal/day. There were no statistically significant differences in macronutrient intakes, especially polyunsaturated/monounsaturated/saturated (P/M/S) fat intake ratio between the baseline (1:0.96:0.72) and the 3-year follow-up (1:1.01:0.74) data. Also, there were no significant differences in total energy expenditure and the proportions of smoking and drinking between the baseline and the 3-year follow-up data (data not shown).

**Table 1:** Clinical characteristics, inflammatory markers, brachial-ankle pulse wave velocity, lipid peroxides, and adhesion molecules at baseline and at the 3-year follow-up

	Baseline	3-year follow-up	*P*
Age (year)	45.3 ± 0.88	48.1 ± 0.89	<0.001
Body mass index (kg/m^2^)	24.6 ± 0.32	24.6 ± 0.29	0.953
Waist-to-hip ratio	0.90 ± 0.01	0.91 ± 0.01	0.267
Systolic BP (mmHg)	120.1 ± 1.50	120.8 ± 1.99	0.940
Diastolic BP (mmHg)	74.9 ± 1.35	75.2 ± 1.60	0.954
Triglyceride (mg/dL)^a^	137.8 ± 10.7	138.8 ± 10.7	0.953
Total cholesterol (mg/dL)^a^	192.4 ± 4.61	190.3 ± 4.31	0.987
HDL cholesterol (mg/dL)^a^	51.5 ± 1.87	45.3 ± 1.50	<0.001
LDL cholesterol (mg/dL)^a^	114.9 ± 4.73	118.8 ± 3.95	0.119
Glucose (mg/dL)^a^	93.9 ± 1.55	96.0 ± 1.72	0.117
Insulin (μU/mL)^a^	8.34 ± 0.43	8.04 ± 0.48	0.581
HOMA-IR^a^	1.94 ± 0.11	1.90 ± 0.12	0.946
hs-CRP (mg/L)^a^	1.23 ± 0.25	0.84 ± 0.08	0.941
Serum IL-6 (pg/mL)^a^	4.83 ± 0.50	4.17 ± 0.46	0.130
White blood cells (×10^9^/L)^a^	6.16 ± 0.31	5.77 ± 0.17	0.639
Adiponectin (μg/mL)^a^	5.27 ± 0.31	5.39 ± 0.27	0.224
ba-PWV (cm/s)^a^	1,340.3 ± 27.5	1,357.2 ± 29.5	0.264
8-epi-PGF_2α_ (pg/mg creatinine)^a^	1,353.8 ± 73.7	1,384.6 ± 67.5	0.838
Malondialdehyde (nmol/mL)^a^	9.92 ± 0.31	12.4 ± 0.47	<0.001
Oxidized LDL (U/L)^a^	34.0 ± 1.46	43.0 ± 1.89	<0.001
sICAM-1 (pg/mL)	193.3 ± 7.65	193.5 ± 9.38	0.658
sVCAM-1 (pg/mL)	676.5 ± 46.9	749.7 ± 65.4	0.043
P-selectin (pg/mL)	33.9 ± 1.65	37.1 ± 2.20	0.153

*P*-values derived from paired *t*-test with the Wilcoxon signed-rank test

*HOMA-IR* {fasting insulin (μIU/mL) × fasting glucose (mmol/L)}/22.5

^a^Mean ± SE tested by logarithmic transformation

### Multivariate statistical analysis and identification of plasma metabolites

**Fig. 1:**
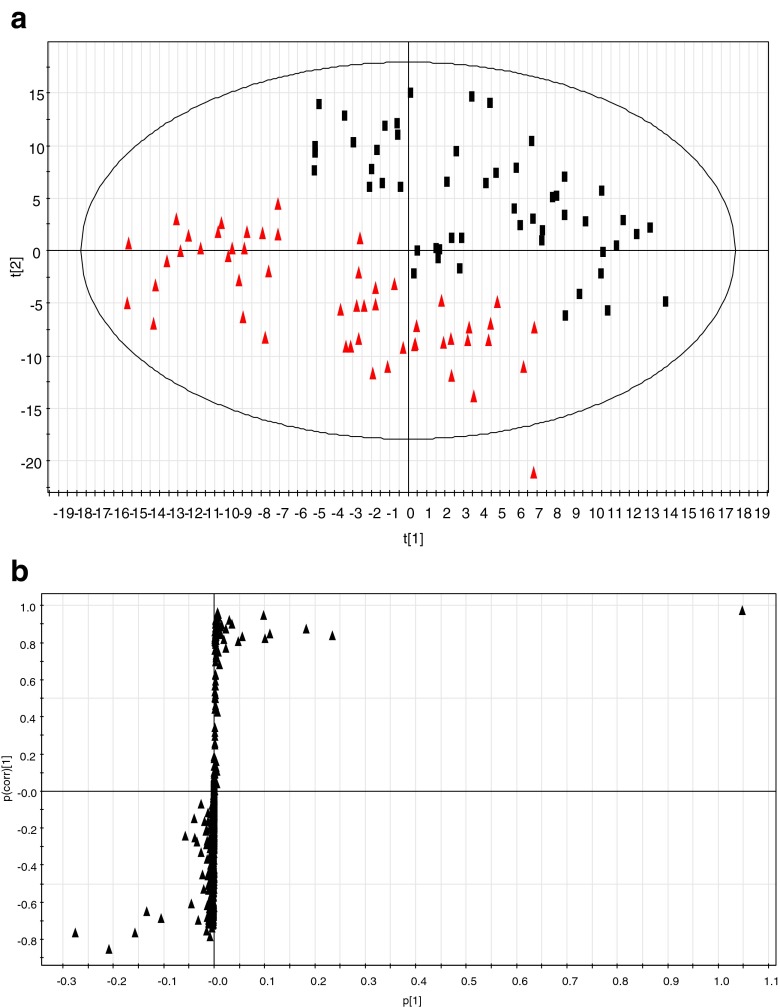
**a** Score plots from PLS-DA models classifying healthy men at baseline (*filled square*) and at 3-year follow-up (*filled triangle*). **b** *S*-plot for covariance [*p*] and reliability correlation [*p*(corr)] from PLS-DA models

The MS data of plasma metabolites obtained from healthy men at baseline and at 3-year follow-up were applied to a PLS-DA score plot (Fig. 1a). The first two-component PLS-DA score plots of the plasma metabolites showed distinct clustering for each group of healthy men at baseline and at 3-year follow-up. Both groups could be clearly differentiated from each other by the primary component t(1) or the secondary component t(2) based on the model with R^2^X (cum) and R^2^Y (cum) values of 0.391 and 0.990, respectively, indicating the goodness of fit of the data. The Q^2^Y (cum) value of 0.818 estimated the predictive ability of the model. In addition, the PLS-DA models were validated using a permutation test and indicated an R^2^Y intercept value of 0.0971 and a Q^2^Y intercept value of 0.0113. To identify the metabolites contributing to the discrimination between the baseline and the 3-year follow-up data, *S*-plots of *p*(1) and *p*(corr)(1) were generated using centroid scaling (Fig. 1b). The *S*-plots revealed that the metabolites with higher or lower *p*(corr) values served as the more relevant ions for discriminating between the two groups.

Among the 813 metabolites in the plasma, the metabolites that play an important role in determining age-related changes after the 3-year follow-up were selected according to their variable importance in the projection (VIP) scores. The normalized intensities of whole metabolites were statistically analyzed by a nonparametric *t*-test; the metabolites with significant differences between the baseline and 3-year follow-up data were included. Thus, 17 metabolites were selected based on their VIP values and independent *t*-tests. Finally, 15 metabolites were identified (two were unknown). The results of the UPLC-Q-TOF analysis are shown in Table 2.

**Table 2:** Identification of plasma metabolites at baseline and at 3-year follow-up

Identity	Formula [M + H]^+^	Exact mass (M + H)	Mass error (mDa)	Normalized peak intensities (mean ± SE)		Fold change^a^ (vs. controls)	*P* ^b^	VIP
				Baseline	Follow-up			
l-Valine	C_5_H_11_NO_2_	118.0789	−6.0	13.0 ± 0.45	15.3 ± 0.53	1.140	0.001	0.2058
Pyrroline hydroxycarboxylic acid	C_5_H_7_NO_3_	130.0425	−6.4	3.30 ± 0.10	2.54 ± 0.10	0.737	<0.001	0.2809
l − Tryptophan	C_11_H_12_N_2_O_2_	205.0898	−7.6	32.1 ± 1.56	31.4 ± 1.64	0.944	0.704	0.8036
Linoleyl carnitine	C_25_H_45_NO_4_	424.3348	−4.4	25.6 ± 1.09	21.9 ± 1.29	0.845	0.009	1.4369
LysoPC (16:0)	C_24_H_50_NO_7_P	496.3324	−6.0	718.7 ± 16.2	778.1 ± 27.1	1.064	0.028	6.2029
LysoPC (18:0)	C_26_H_54_NO_7_P	524.3637	−6.2	545.9 ± 15.0	574.0 ± 17.0	1.043	0.231	4.6709
LysoPC (18:2)	C_26_H_50_NO_7_P	520.3325	−5.9	467.8 ± 11.4	510.6 ± 14.4	1.081	0.004	4.6890
LysoPC (18:3)	C_26_H_48_NO_7_P	518.3168	−6.2	12.5 ± 0.66	14.8 ± 0.98	1.152	0.068	0.4923
LysoPC (20:4)	C_28_H_50_NO_7_P	544.3325	2.5	54.7 ± 3.56	66.1 ± 6.38	1.175	0.021	1.4186
LysoPC (20:5)	C_28_H_48_NO_7_P	542.3168	−0.7	39.1 ± 1.52	45.4 ± 1.32	1.134	0.002	1.1194
LysoPC (22:5)	C_30_H_52_NO_7_P	570.3481	−8.3	1.88 ± 0.18	2.56 ± 0.22	1.272	0.002	0.0565
LysoPC (22:6)	C_30_H_50_NO_7_P	568.3325	3.6	48.3 ± 1.36	53.0 ± 1.16	1.083	0.007	0.5201
LysoPE (18:0)	C_23_H_48_NO_7_P	482.3168	−7.4	8.88 ± 0.50	10.7 ± 0.43	1.176	0.004	0.2748
LysoPE (22:6)	C_27_H_44_NO_7_P	526.2855	−5.8	28.1 ± 0.77	31.5 ± 0.98	1.107	0.003	0.5602
Total LysoPC	–	–	–	8.59 ± 1.23	6.51 ± 0.83	0.713	0.001	–
Unknown 1	–	417.3290	−7.3	–	–	0.100	–	0.5015
Unknown 2	–	585.2634	−8.5	–	-	1.720	-	0.5597

^a^Mean ± SE calculated by the mean of intensity of each metabolite from cases by the mean of intensity of each metabolite from controls

^b^ *P*-values derived from paired *t*-test with the Wilcoxon signed-rank test

Eight plasma metabolites, including l-valine, lysophosphatidyl cholines (lysoPCs) containing C16:0, C18:2, C20:4, C20:5, C22:5, and C22:6, and lysophosphatidyl ethanolamines (lysoPEs) containing C18:0 and C22:6, showed significant increases at 3-year follow-up, whereas three metabolites, including pyrroline hydroxycarboxylic acid, linoleyl carnitine, and total lysoPCs, showed decreased levels. Linoleyl carnitine and lysoPCs containing C16:0, C18:0, C18:2, C20:4, and C20:5 (with VIP values >1.0, indicating a high relevance to the difference between the sample groups) were the major plasma metabolites contributing to the discrimination between the baseline and 3-year follow-up data on the PLS-DA score plot (Table 2). In particular, lysoPC 16:0 with a VIP value of 6.2029 served as the most important plasma metabolite for evaluating the differences between the baseline and 3-year follow-up data.

### Relationship between the changes in the major plasma metabolite levels

The changes in lysoPC 16:0 levels were positively correlated with the changes in lysoPC 18:0 (*r* = 0.624, *P* < 0.001), lysoPC 18:2 (*r* = 0.620, *P* < 0.001), and lysoPC 20:5 (*r* = 0.414, *P* = 0.003) levels. The changes in lysoPC 18:0 were positively correlated with the changes in lysoPC 18:2 (*r* = 0.773, *P* < 0.001) and lysoPC 20:5 (*r* = 0.600, *P* < 0.001). The changes in lysoPC 18:2 were positively correlated with the changes in lysoPC 20:4 (*r* = 0.325, *P* = 0.023) and lysoPC 20:5 (*r* = 0.395, *P* = 0.005). The changes in linoleyl carnitine were positively correlated with the changes in pyrroline hydroxycarboxylic acid (*r* = 0.464, *P* = 0.001).

### Relationship between the changes in levels of lysoPC 16:0, arterial stiffness, lipid peroxides, inflammatory markers, and adhesion molecules

**Fig. 2:**
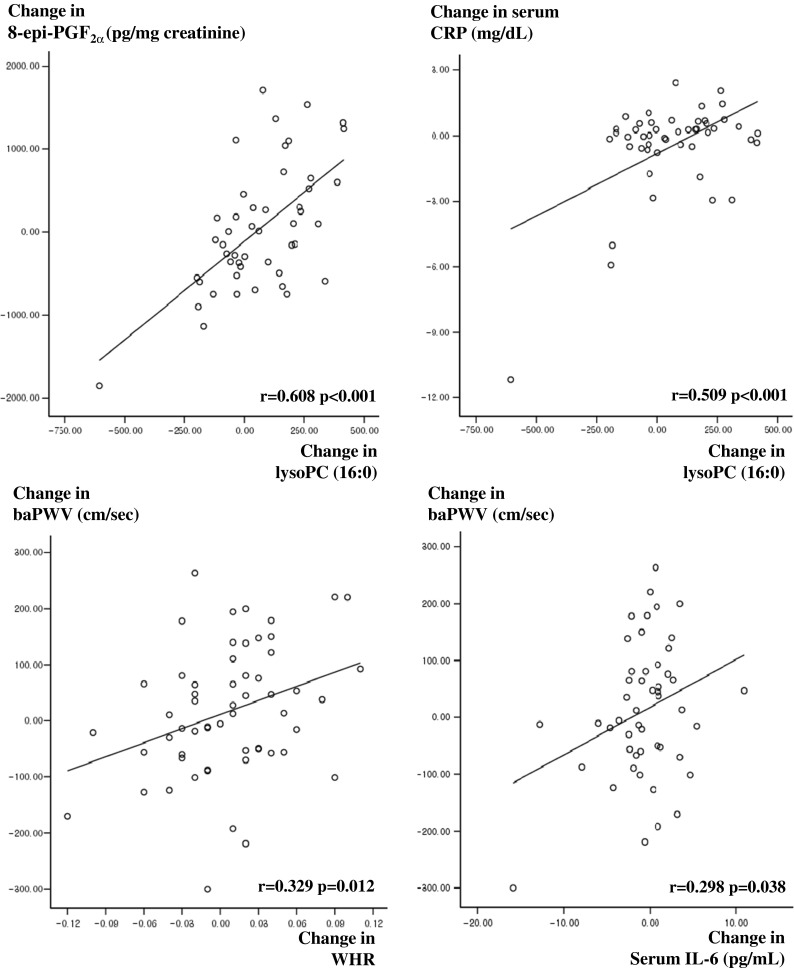
Relationship of the changes in lysoPC (16:0) levels, WHR, and IL-6 with the changes in 8-epi-PGF_2α_, CRP, and ba-PWV in healthy men after 3 years. Tested by Pearson correlation analysis. *r*, correlation coefficient

The changes in lysoPC 16:0 between the baseline and 3-year follow-up data were positively correlated with the changes in 8-epi-PGF_2α_ (*r* = 0.608, *P* < 0.001) (Fig. 2), MDA (*r* = 0.413, *P* = 0.004), hs-CRP (*r* = 0.509, *P* < 0.001) (Fig. 2), IL-6 (*r* = 0.497, *P* = 0.001), and ba-PWV (*r* = 0.283, *P* = 0.049). Additionally, decreased HDL cholesterol was significantly associated with increased levels of lysoPC 20:5 (*r* = −0.286, *P* = 0.049) and lysoPC 22:6 (*r* = −0.422, *P* = 0.003) among the 15 endogenous metabolites, which were maintained after adjusting for changed values of age, BMI, LDL cholesterol, and triglyceride (*r* = −0.295, *P* = 0.052; *r* = −0.466, *P* = 0.001, respectively). The changes in ba-PWV were positively correlated with the changes in waist-to-hip ratio (WHR) (*r* = 0.329, *P* = 0.012) (Fig. 2), ox-LDLs (*r* = 0.312, *P* = 0.023), MDA (*r* = 0.302, *P* = 0.024), hs-CRP (*r* = 0.329, *P* = 0.013), IL-6 (*r* = 0.298, *P* = 0.038) (Fig. 2), and P-selectin (*r* = 0.345, *P* = 0.034). The changes in hs-CRP were positively correlated with the changes in 8-epi-PGF_2α_ (*r* = 0.541, *P* < 0.001) (Fig. 2), ox-LDLs (*r* = 0.296, *P* = 0.033), and IL-6 (*r* = 0.635, *P* < 0.001). Additionally the changes in IL-6 were also positively correlated with the changes in WHR (*r* = 0.287, *P* = 0.046) and 8-epi-PGF_2α_ (*r* = 0.311, *P* = 0.031). Furthermore, the ratio of total calorie intake to total calorie expenditure (TCI/TEE) was closely related with the changes in WHR (*r* = 0.450, *P* < 0.001).

### Association of the levels of inflammatory markers, lipid peroxides, adhesion molecules, and metabolites with the changes in arterial stiffness

Because of the close relationship between the changes in levels of lysoPC 16:0, arterial stiffness, lipid peroxides, inflammatory markers, and adhesion molecules, we divided our subjects into two groups according to the changes in arterial stiffness from baseline to 3-year follow-up (decreased ba-PWV vs. increased ba-PWV groups). As ba-PWV was highly associated with blood pressure (systolic and diastolic), we adjusted for blood pressure when we compared the values between the groups. At baseline, men in the increased ba-PWV group (*n* = 30) had lower hs-CRP levels (*P* = 0.039) than those in the decreased ba-PWV group had. After 3 years, men in the decreased ba-PWV group (*n* = 27) showed a significant reduction in ba-PWVs (*P* < 0.001) and serum IL-6 (*P* = 0.038) levels and significant increases in the levels of MDA (*P* = 0.032) and ox-LDLs (*P* = 0.009) (Table 3). Men in the increased ba-PWV group showed significant increases in WHR (*P* = 0.025) and levels of ba-PWV (*P* < 0.001), MDA (*P* < 0.001), ox-LDLs (*P* = 0.002), and P-selectin (*P* = 0.002) after 3 years. In addition, the changes in WHR (*P* = 0.043), ba-PWV (*P* < 0.001), IL-6 (*P* = 0.038), 8-epi-PGF_2α_ (*P* = 0.004), MDA (*P* = 0.027), and P-selectin (*P* = 0.004) levels were significantly different between the two ba-PWV groups after adjustment for changes of blood pressure (Table 3). Additionally, changes in lysoPC 16:0 levels were significantly different between the decreased and increased ba-PWV groups (−14.7 ± 50.7 vs. 106.4 ± 29.5; *P* = 0.049) after the adjustment. There were no significant changes in other plasma metabolite levels between the ba-PWV groups before or after adjustment for baseline values (data not shown).

**Table 3:** Inflammatory markers, lipid peroxides, and adhesion molecules according to changes in arterial stiffness at baseline and at 3-year follow-up

	Controls (PWV decreased) (*n* = 27)	*P* ^a^	Cases (PWV increased) (*n* = 30)	*P* ^a^	*P* ^b^
Body mass index (kg/m^2^)					
Baseline	23.9 ± 0.47	0.343	25.3 ± 0.41	0.251	0.094
Follow-up	24.1 ± 0.35		25.1 ± 0.44		0.235
Change	0.16 ± 0.27		−0.16 ± 0.13		0.194
Waist-to-hip ratio					
Baseline	0.91 ± 0.01	0.592	0.89 ± 0.01	0.025	0.247
Follow-up	0.90 ± 0.01		0.91 ± 0.01		0.332
Change	−0.01 ± 0.01		0.02 ± 0.01		0.043
ba-PWV (cm/s)					
Baseline^a^	1,385.9 ± 49.7	<0.001	1,299.3 ± 25.4	<0.001	0.096
Follow-up^a^	1,306.1 ± 50.6		1,403.3 ± 31.4		0.118
Change	−79.9 ± 13.7		104.0 ± 13.0		<0.001
White blood cells (×10^9^/L)					
Baseline^a^	6.71 ± 0.56	0.038	5.67 ± 0.29	0.508	0.088
Follow-up^a^	5.74 ± 0.25		5.81 ± 0.24		0.909
Change	−0.97 ± 0.50		0.14 ± 0.26		0.043
hs-CRP (mg/L)					
Baseline^a^	1.84 ± 0.51	0.039	0.71 ± 0.11	0.040	0.005
Follow-up^a^	0.73 ± 0.09		0.94 ± 0.13		0.617
Change	−1.11 ± 0.53		0.23 ± 0.17		0.007
Serum IL-6 (pg/mL)					
Baseline^a^	5.40 ± 0.82	0.038	4.28 ± 0.60	0.757	0.301
Follow-up^a^	3.37 ± 0.43		4.94 ± 0.78		0.226
Change	−2.03 ± 1.01		0.67 ± 0.57		0.038
8-epi-PGF_2α_ (pg/mg creatinine)					
Baseline^a^	1,307.0 ± 111.1	0.399	1,394.5 ± 99.6	0.039	0.985
Follow-up^a^	1,182.9 ± 73.1		1,559.4 ± 99.3		0.003
Change	−124.1 ± 115.6		164.9 ± 144.0		0.027
MDA (nmol/mL)					
Baseline^a^	10.2 ± 0.46	0.032	9.68 ± 0.42	<0.001	0.292
Follow-up^a^	11.6 ± 0.55		13.1 ± 0.72		0.165
Change	1.42 ± 0.54		3.46 ± 0.67		0.027
Oxidized LDL (U/L)					
Baseline^a^	33.6 ± 2.08	0.009	34.3 ± 2.08	0.002	0.951
Follow-up^a^	41.4 ± 2.07		44.7 ± 3.21		0.954
Change	7.81 ± 2.66		10.4 ± 2.60		0.788
sICAM-1 (pg/mL)					
Baseline	181.7 ± 10.6	0.526	201.7 ± 10.6	0.676	0.290
Follow-up	187.6 ± 15.2		197.8 ± 12.1		0.615
Change	5.91 ± 10.7		−3.96 ± 5.81		0.555
sVCAM-1 (pg/mL)					
Baseline	698.6 ± 74.4	0.335	660.5 ± 61.5	0.138	0.455
Follow-up	755.5 ± 104.5		745.6 ± 85.8		0.829
Change	56.9 ± 66.8		85.1 ± 65.4		0.659
P-selectin (pg/mL)					
Baseline	36.3 ± 2.92	0.342	32.2 ± 1.87	0.002	0.188
Follow-up	33.8 ± 3.06		39.5 ± 3.04		0.183
Change	−2.53 ± 2.34		7.33 ± 2.67		0.004

^a^Mean ± SE tested by logarithmic transformation. Tested by general linear model tests (mixed model tests) with adjustment

^a^ *P*-values within a group derived after adjusting for changed SBP and DBP

^b^ *P*-values between groups derived after adjusting for changed SBP and DBP

*ba-PWV* brachial-ankle pulse wave velocity, *MDA* malondialdehyde

## Discussion

Using a metabolomics approach based on UPLC/Q-TOF MS, we identified 15 endogenous metabolites that showed age-related changes in middle-aged men. Among these metabolites, lysoPCs containing C16:0, C18:0, C18:2, C20:4, and C20:5 and linoleyl carnitine were the six major metabolites contributing to the discrimination between the baseline and 3-year follow-up. Various species of lysoPC are defined by fatty acid chain length and degree of saturation, which may translate into different physical and biological properties (Loftus et al. 2008). Although lysoPC levels obviously increase in aging rats (Fu et al. 2011), this important issue is largely unexplored in humans. In our study, among the six major metabolites identified by mass spectrometric analysis, lysoPC 16:0 was found to be the most important plasma metabolite for evaluating aging-related changes. Additionally, the changes in lysoPC 16:0 levels were strongly positively correlated with the changes in levels of 8-epi-PGF_2α_, a reliable marker of oxidative stress (Wolfram et al. 2005; Vassalle et al. 2004), MDA, hs-CRP, IL-6, and ba-PWVs. This result suggests that age-related changes in lysoPC 16:0 levels in middle-aged men could contribute to lipid peroxidation, the activation of a proinflammatory phenotype, and arterial stiffness. Furthermore, increases in lysoPC 16:0 levels from the baseline to the 3-year follow-up data were significantly greater in subjects with increased ba-PWVs. Pulse wave velocity is an established index of arterial stiffness (Tomiyama and Yamashina 2010), and ba-PWVs show similar characteristics to those of central aortic PWV (Tsuchikura et al. 2010).

Arterial stiffness, one of the most significant manifestations of vascular aging (Lakatta and Levy 2003; Lakatta 2003), can result in increased systolic blood pressure (Dao et al. 2005; O’Rourke and Hashimoto 2007). This is a condition that can worsen with age, even in healthy individuals without CVD. In addition, the presence of CVD risk factors such as obesity may accelerate the vascular changes that result in arterial stiffness (Zieman et al. 2005). However, in our study, we did not find significant changes overall in ba-PWV and WHR in subjects during the 3-year follow-up. When we subdivided study subjects according to the changed levels of ba-PWV, we found interesting results. Men in the subgroup of increased ba-PWVs showed significant increases in abdominal obesity, which were correlated with increases in IL-6 and the ratio of TCI/TEE (the ratio of total calorie intake to total calorie expenditure). Additionally, changes in WHR, MDA, hs-CRP, IL-6, 8-epi-PGF_2α_, and P-selectin levels were significantly different between subjects with decreased and increased ba-PWVs. However, the changes in MDA together with those in ox-LDL were in the same direction in both ba-PWV sub-groups (increased in both) which may need to be further studied as to whether this phenomenon occurs independent of ba-PWV change or not. Actually, the mechanisms underlying arterial stiffening remain to be elucidated, but the fact that changes in ba-PWV between the baseline and 3-year follow-up data were positively correlated with the changes in WHR, ox-LDLs, MDA, hs-CRP, and IL-6 levels suggest that changes in oxidative stress, proinflammation, or abdominal obesity could play an important role, in part, in accelerating arterial stiffness. Recently, Brinkley et al. (2009) suggested that ox-LDL levels may be related to the pathogenesis of arterial stiffness, independent of other CVD risk factors.

LysoPC constitutes only 1–5 % of the total PC content of non-ox-LDL; however, as much as 40–50 % of the PC contained within the LDL molecule is converted to lysoPC during LDL oxidation (Matsumoto et al. 2007). A saturated fatty acid or a monounsaturated fatty acid predominates in the sn-1 position of the phospholipid (Stafforini et al. 2006). The generation of free radicals as a result of oxidative stress can activate phospholipase A_2_ (PLA_2_), which hydrolyzes phosphatidylcholine (PC) to produce lysoPCs (Steinbrecher and Pritchard 1989). Production of α-palmitoyl-lysoPC (C16:0) can stimulate endothelial cells to express adhesion molecules and release cytokines (Takabe et al. 2004; Kume et al. 1992; Murohara et al. 1996; Zhu et al. 1997; Liu-Wu et al. 1998; Rong et al. 2002). In fact, IL-6 was found to be induced by α-palmitoyl-lysoPC treatment in human umbilical vein endothelial cells. In line with this result, our study showed a significant age-related increase in both ox-LDL and lysoPC 16:0 levels and a positive relationship between the changes in lysoPC 16:0 and IL-6 levels.

PLA_2_, including secretory PLA_2_ (sPLA_2_) and lipoprotein-associated phospholipase (Lp-PLA_2_), hydrolyzes PC, simultaneously generating one molecule of lysoPC and one molecule of arachidonic acid, a precursor of eicosanoids such as prostaglandins and leukotrienes (Matsumoto et al. 2007). Radical peroxidation of arachidonic acid results in a family prostaglandin F_2_-isomers called F_2_ isoprostanes (Voss and Siem 2006). One such F_2_-isoprostane is 8-epi-PGF_2α_, a sensitive marker for oxidative stress (Wolfram et al. 2005; Vassalle et al. 2004) that is probably released into biological fluids through a phospholipase-mediated pathway and consequently excreted in urine. In this study, changes in lysoPC 16:0 were closely and positively associated with changes in urinary 8-epi-PGF_2α_ concentrations. Additionally, changes in lysoPC 16:0 and 8-epi-PGF_2α_ were closely related with changes in CRP concentrations.

LysoPCs, representing 5–20 % of the total plasma phospholipids (Nelson 1967), are also formed by the action of lecithin cholesterol acyltransferase (LCAT) in plasma (Kougias et al. 2006). Human LCAT releases lysoPC 20:4 and 22:6 from the sn-1 position of PC (Liu et al. 1998). In plasma, up to 80 % of the lysoPC is found in the non-lipoprotein fraction, in which albumin is considered to be the main lipid-binding protein (Ojala et al. 2006). Unsaturated lysoPCs are mainly associated with albumin rather than lipoproteins (Croset et al. 2000). In our study, we observed a positive relationship between the changes in levels of lysoPC 16:0 and those of lysoPCs containing C18:0, C18:2, and C20:5, which could reflect an alternative source of lysoPC C16:0 production in addition to ox-LDLs.

In our study, a large number of metabolite markers were detected by UPLC-MS; however, most of these metabolite markers remain unidentified. Unlike gas chromatography–mass spectrometry, for which large databases exist, the use of liquid chromatography–mass spectrometry-based techniques for metabolomics research is still in its infancy, and the databases of endogenous biomolecules have not yet been constructed (Williams et al. 2006). Despite this limitation, using a UPLC-Q-TOF MS-based metabolomics strategy and multivariate data analysis, our study identified a cluster of age-associated changes in plasma metabolites that included six major metabolites: lysoPCs containing C16:0, C18:0, C18:2, C20:4, and C20:5 and linoleyl carnitine. Among these six major metabolites, lysoPC 16:0 served as the most important plasma metabolite for evaluating age-related differences between the baseline and 3-year follow-up data. Additionally, the changes in lysoPC 16:0 levels were positively correlated with the changes in levels of lipid peroxides, proinflammatory markers, and ba-PWV. These results suggest that increases in lysoPC 16:0 can be explored further as a potential marker for lipid peroxidation, the activation of a proinflammatory phenotype, and arterial stiffness related to aging.
